# Development of a Theoretically Informed Web-Based Mind-Body Wellness Intervention for Patients With Primary Biliary Cholangitis: Formative Study

**DOI:** 10.2196/29064

**Published:** 2021-10-08

**Authors:** Makayla Watt, John C Spence, Puneeta Tandon

**Affiliations:** 1 University of Alberta Edmonton, AB Canada

**Keywords:** liver disease, meditation, yoga, breathwork, behavior theory, COM-B model, behaviour change wheel, behaviour change taxonomy, internet, digital

## Abstract

**Background:**

Mind-body interventions have the potential to positively impact the symptom burden associated with primary biliary cholangitis (PBC). Interventions are more likely to be effective if they are informed by a theoretical framework. The Behaviour Change Wheel (BCW) and the behaviour change technique taxonomy version 1 (BCTv1) provide frameworks for intervention development.

**Objective:**

This study describes how theory has guided the development of a 12-week multicomponent mind-body wellness intervention for PBC.

**Methods:**

The steps involved in developing the BCW intervention included specifying the target behavior; explaining barriers and facilitators using the Capability, Opportunity, Motivation, and Behaviour and the theoretical domains framework; identifying intervention functions to target explanatory domains; and selecting relevant behavior change techniques to address intervention functions. Qualitative data from patients with inflammatory bowel disease using an earlier version of the program and feedback from a PBC patient advisory team were used to guide intervention development.

**Results:**

Barriers and facilitators to intervention participation associated with capability, opportunity, and motivation were identified. Intervention functions and behavior change techniques were identified to target each barrier and facilitator.

**Conclusions:**

The Peace Power Pack PBC intervention was developed to help individuals with PBC manage their symptom burden. The theoretical frameworks employed in this intervention provide direction on targeting antecedents of behavior and allow standardized reporting of intervention components.

**Trial Registration:**

ClinicalTrials.gov NCT04791527; https://clinicaltrials.gov/ct2/show/NCT04791527

## Introduction

Primary biliary cholangitis (PBC) is a female predominant chronic liver disease estimated to affect between 9000 and 11,000 Canadians [1]. Despite the relatively low prevalence of PBC, global incidence and prevalence rates have been reported to be on the rise [1]. PBC is associated with symptoms including pruritus and fatigue, which can lead to social isolation and emotional dysfunction [1-3]. Fatigue, defined as a persistent state of exhaustion, inability to perform usual routines, and a decreased capacity for physical and mental work, has been reported as the most common and debilitating among these symptoms [4-7]. Individuals with PBC also commonly experience a low health-related quality of life (HRQOL), with 1 study concluding that 35% of individuals with PBC had an impaired HRQOL compared to 6% of healthy controls [6]. Current medical therapies are ineffective at improving PBC-related symptoms or impacting quality of life [5,6,8]. Building upon the recognized need for novel interventions [6,9], our team was approached by patients and the Canadian PBC Society to develop self-care tools to manage symptom burden. Although to our knowledge, mind-body wellness interventions have never been trialed in PBC, interventions of this nature have been found to improve fatigue and HRQOL in other chronic diseases [10-12].

The use of a clear theoretical framework during the design of an intervention has been associated with increased adherence rates, and sustained changes to health-related behaviors [13-15]. The Behaviour Change Wheel (BCW), a framework synthesized from 19 individual models of behavior, has been used to guide development of several acceptable and effective theory-based interventions [16-18]. At the core of the BCW is the Capability, Opportunity, Motivation and Behaviour (COM-B) model, which describes the key antecedents to the target behavior. The BCW then outlines intervention functions that can be used to facilitate behavior change [16]. This process is further enhanced by the behavior change technique taxonomy version 1 (BCTv1), which details standardized active intervention ingredients that can be implemented to target intervention functions [19]. Optimally, theory would also extend to the evaluation of behavior change and maintenance.

This paper describes how theory has guided the development of a 12-week multicomponent mind-body wellness intervention for PBC (ClinicalTrials.gov NCT04791527) using several theoretical constructs: BCW guidelines [16], the COM-B model [16], the theoretical domains framework (TDF) [20], and the BCTv1 [19]. Development of the intervention involved the following steps, which were informed by the BCW guidelines: (1) specify the target behavior; (2) explain barriers and facilitators to the target behavior by using the COM-B model and the TDF; (3) identify intervention functions to target explanatory domains; and (4) select relevant behavior change techniques to address intervention functions.

## Methods

**Figure 1:**
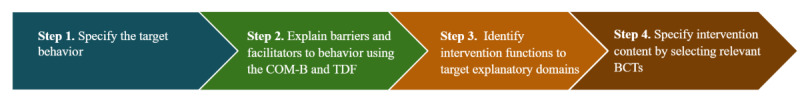
Steps involved in intervention development. BCT: behavior change technique; COM-B: Capability, Opportunity, Motivation and Behaviour; TDF: theoretical domains framework.

The following sections outline the processes (methods) for each of the 4 steps of intervention development. An outline of the 4 steps of intervention development can be found in Figure 1.

### Step 1: Specify the Target Behavior

The target behavior was determined through a review of the literature on adherence to behavioral health interventions, and in consultation with the Canadian PBC Society. 

### Step 2: Explain Barriers and Facilitators to Behavior Using the COM-B and TDF

Domains from the COM-B model and the TDF were selected to explain barriers and facilitators to the target behavior. The COM-B model outlines that for a behavior to occur, an individual must have the capability, opportunity, and motivation to perform the behavior. Capability is composed of psychological capability (knowledge), and physical capability (physical skills); opportunity is composed of physical opportunity (environmental resources) and social opportunity (cultural milieu); and motivation includes reflective motivation (evaluations, plans) and automatic motivation (emotions, impulses) [16]. As the COM-B model provides a relatively general understanding of behavior, the TDF, which outlines 14 processes involved in behavior change, is often used to provide further specification of behavioral determinants [20]. To identify barriers and facilitators driving health-related behavior, we conducted qualitative interviews with individuals who had participated in the previous iteration of the intervention carried out in a separate chronic disease group (ie, individuals with inflammatory bowel disease [IBD]) [21]. Similar to PBC, individuals with IBD experience high rates of fatigue and impaired quality of life [22,23]. These interviews were coded and thematically analyzed by 2 independent coders [21]. A COM-B characteristic and a TDF domain were then identified for each barrier and facilitator of behavior mentioned by those participants.

### Step 3: Identify Intervention Functions to Target Explanatory Domains

Intervention functions were selected to address each barrier and facilitator to behavior. The BCW specifies 9 standardized intervention functions that can be used to address barriers and facilitators to behavior change [16]. The BCW guide then outlines intervention functions that are appropriate for each TDF domain [24]. The web-based nature of the program and characteristics of the target population (ie, chronic fatigue) were considered when selecting intervention functions.

### Step 4: Specify Intervention Content by Selecting Relevant BCTs

Behavior change techniques were selected to allow standardized implementation of intervention functions. Following the procedure outlined by Jennings et al [13] and Tombor et al [25], BCTs were specified for each of the intervention functions identified in step 3. 

## Results

### Results Overview

The Peace Power Pack PBC (PPP_PBC_) intervention was co-developed with a patient advisory team from the Canadian PBC society. The web-based intervention is described in Multimedia Appendix 1. The intervention is 12 weeks in duration with each week featuring: (1) a video detailing a core practice of mindful movement (yoga, tai chi, and low-intensity exercise divided into a standing stream and a chair stream), energizing breathwork practices, and guided meditation (increasing in length from 20-30 minutes over the course of the program); (2) an introductory video describing a weekly positive psychology theme (3-5 minutes); and (3) an interactive positive psychology activity related to the theme for the week (3-5 minutes). All programming is hosted on the investigator’s website [26]. Throughout the duration of the study, participants will receive standardized weekly motivational emails, weekly 10-minute motivational interviewing check-ins, and will be invited to participate in weekly group sessions with fellow participants. The following section outlines the outcomes (results) for each of the 4 steps of intervention development previously outlined.

### Step 1: Specify the Target Behavior

Adherence to the video-based program at least 3 days a week was selected as the primary target behavior, with a gradual increase in the video duration over the course of the 12 weeks. Based on feedback from the Canadian PBC society, this target behavior was chosen with the intent to balance the intervention dose with likelihood of adherence. Available evidence suggests that higher levels of adherence to behavioral health interventions leads to improved outcomes in a dose-dependent manner [27]. High levels of fatigue in individuals with PBC have been associated with a decreased sense of self-efficacy for a particular behavior [28] and inability to adhere to a target could lead to further reductions in self-efficacy. To ensure participants are aware of the anticipated study commitment, the target will be advertised to participants interested in enrollment.

### Step 2: Explain Barriers and Facilitators to Behavior Using the COM-B and TDF

A comprehensive list of barriers and facilitators, along with the associated COM-B and TDF domains is provided in Table 1. The most common barriers to program participation described by the individuals with IBD were difficulty fitting the program into daily routine, and finding that the movement portion of the program was not matched with the ability level [21]. Perceived facilitators to program participation included accessible presentation of content on the host website and contact with program facilitators/fellow participants. Of the 14 domains of the TDF, 9 were associated with barriers and facilitators to intervention participation: behavior regulation, physical skills, environmental context and resources, memory attention and decision processes, social influences, goals, beliefs about capabilities, beliefs about consequences, and reinforcement. The most common TDF domains were social influences (check-ins with program facilitators and other participants enhancing accountability), and behavioral regulation (fitting the program into daily routine).

**Table 1:** Use of behavior change techniques in developing an intervention for people living with primary biliary cholangitis.

Enabler	Barrier	COM-B^a^/TDF^b^/IF^c^	Behavior change technique	Implementation of a behavior change technique
Interactions with program facilitators enhanced accountability		COM-B: reflective motivation TDF: goals IF: persuasion	1.5 Review behavior goal(s) 1.6 Discrepancy between current behavior and goal 3.1 Social support (unspecified)	1.5 Weekly adherence vs target adherence goal were discussed during check in 1.6 Weekly adherence vs target adherence goal were discussed during check in 3.1 Weekly check ins employed motivational interviewing techniques to support program adherence
Able to integrate in everyday routine	Difficulty integrating program into daily routine	COM-B: psychological capability TDF: behavioral regulation IF: enablement, persuasion	1.4 Action planning (Future consideration 1.6 Discrepancy between current behavior and goal 2.2 Feedback on behavior 15.3 Focus on past success (self-belief)	1.4 In week 1, participants watched an interactive video prompting them to plan their performance of the target behavior (adherence to the program at or above the set minimum adherence goal). This included committing to a personal adherence goal at or above the set minimum, and writing down (1) potential obstacles to meeting their adherence goal; and (2) actions that could be taken to avoid or overcome these obstacles. 1.6 The host website recorded weekly participation (indicated by accessed content). At the top of the website, the user’s current weekly participation was presented beside the user’s adherence goal. 2.2 The host website recorded weekly participation (indicated by accessed content). At the top of the website, the user’s current weekly participation was presented. 15.3 In week 1, participants watched an interactive video that prompted them to think about instances in which they successfully adhered to a goal.
Access to accommodations to physical activity program where needed	Insufficient access to accommodation to physical activity program	COM-B: physical capability TDF: skills IF: enablement	4.1 Instruction on how to perform a behavior 6.1 Demonstration of the behavior	4.1 Instruction for accommodations were provided 6.1 Demonstration of accommodations were provided
Interaction with others in program associated with increased motivation		COM-B: social opportunity TDF: social influences IF: persuasion, modeling	3.1 Social support (unspecified)	3.1 Participants were invited to weekly live group sessions in which they had the opportunity to participate in program practices with peers
Desire to feel better		COM-B: reflective motivation TDF: goals IF: persuasion	5.1 Information about health consequences 5.2 Information about emotional consequences	5.1 Introductory videos provided information about health consequences associated with participating in the program 5.2 Introductory videos provided information about health consequences associated with participating in the program
	Difficult to participate when feeling unwell due to disease	COM-B: physical capability, psychological capability TDF: environmental context and resources IF: environmental restructuring	12.1 Restructuring of the physical environment	12.1 Short meditations were provided that could be completed when individuals are not feeling as well 12.1 All mindful movement was low intensity
Able to navigate website	Difficulty navigating website	COM-B: psychological capability TDF: memory attention and decision processes IF: training	4.1 Instruction on how to perform a behavior 6.1 Demonstration of the behavior (comparison of a behavior)	4.1 Individuals received an introduction to the online platform via zoom, in which the research assistant provided instruction on accessing the intervention. Written instructions were also forwarded to all participants in an email. 6.1 Individuals received an introduction to the online platform via zoom in which the research assistant demonstrated accessing the intervention
Web-based format enhanced accessibility		COM-B: psychological capability TDF: environmental context and resources IF: environmental restructuring	12.1 Restructuring the physical environment	12.1 Web-based format was maintained
	Physical movement was too difficult	COM-B: physical capability TDF: physical skills IF: enablement, training, environmental restructuring	4.1 Instruction on how to perform a behavior 6.1 Demonstration of the behavior (comparison of a behavior) 12.1 Restructuring of the physical environment	4.1 Within each stream, the mindful movement videos featured description of how to perform each specific posture/exercise 6.1 Within each stream, the mindful movement videos featured demonstration of how to perform each specific posture/exercise 12.1 Two streams of mindful movement were implemented, which were differentiated by difficulty
	Physical movement was not difficult enough	COM-B: reflective motivation TDF: beliefs about capabilities IF: environmental restructuring	12.1 Restructuring of the physical environment	12.1 Two streams of mindful movement were implemented, which were differentiated by difficulty
Feeling better/good after participating in intervention	Uncertain about benefit	COM-B: reflective motivation TDF: beliefs about consequences, reinforcement IF: persuasion	5.1 Information about health consequences 5.2 Information about emotional consequences 9.1 Credible source	5.1 Introductory videos provided information about health consequences associated with participating in the program 5.2 Introductory videos provided information about health consequences associated with participating in the program 9.1 Introductory videos featured health care professionals discussing potential benefits associated with participating in the program
	Fear of getting injured during physical activity	COM-B: reflective motivation TDF: beliefs about consequences IF: education, environmental restructuring	9.1 Credible source 12.1 Restructuring of the physical environment	9.1 Welcome video featured a health care professional explaining that mindful movement was designed to be safe for PBC. 12.1 Various streams of mindful movement were available, separated by difficulty. Adaptations were available within mindful movement.
Repetition in physical activity program helped build routine		COM-B: psychological capability TDF: memory, attention, and decisional processes IF: enablement	8.3 habit formation	8.3 Routine varied but structure was conveyed through repetition of the same type of activity from week to week (eg, 1 day of each week was dedicated to a breath program, 1 day a flow day)

^a^COM-B: Capability, Opportunity, Motivation and Behaviour.

^b^TDF: theoretical domains framework.

^c^IF: intervention functions.

### Step 3: Identify Intervention Functions to Target Explanatory Domains

The intervention functions persuasion, environmental restructuring, and education were used to target theoretical domains relating to motivation. The intervention functions persuasion, enablement, training, and environmental restructuring were selected to target theoretical domains related to capability, and the intervention functions persuasion and modelling were selected to target theoretical domains related to opportunity. See Table 1 for a full outline of the intervention functions selected for each domain**. **

### Step 4: Specify Intervention Content by Selecting Relevant BCTs

The comprehensive list of selected BCTs along with a description of how they were operationalized can be found in Table 1. Examples of how selected BCTs were translated into each of the general intervention components are detailed in the following. 

#### Implementation of BCTs Into Core Practice

To address the behavior barrier “physical movement was too difficult,” the BCTs “including instructions on how to perform a behaviour,” “demonstration of the behaviour,” and “restructuring of the physical environment” were employed. These were operationalized by including short videos to describe and demonstrate each exercise featured in the mindful movement routines, and through restructuring the program to include participant choice between a chair versus a standing stream of mindful movement. 

#### Implementation of BCTs Into Positive Psychology 

The BCTs “action planning” and “focus on past successes” were integrated into the positive psychology portion of the program to help address the barrier “integrating the program into daily routine.” Specifically, an interactive positive psychology activity at the beginning of the program was created to prompt participants to set their adherence goal, schedule their behavior, consider potential barriers and facilitators to behavior, and think about past successes with behavior change. 

#### Implementation of BCTs Into Weekly Communications

To address the behavior facilitator “interactions with others enhances accountability,” we selected the BCTs “social support (unspecified),” “review behaviour goals,” and “discrepancy between current behaviour and goal.” During the weekly phone check-ins, a program facilitator will implement these BCTs by providing social support through brief weekly motivational interviewing touchpoints, revisiting the participant’s initial goals, and discussing weekly adherence versus initial adherence goals. 

## Discussion

### Principal Findings

The PPP_PBC_ intervention was developed to provide individuals with PBC a tool to help better manage their symptom burden. The intervention was designed to optimize participation by enhancing a participant’s physical capability (ie, enable participation in a stream of mindful movement), psychological capability (ie, enable self-regulation), automatic motivation (ie, help participants build a routine), reflective motivation (ie, building intention to participate in wellness practices), and social opportunity (ie, connect with peer models). Owing to the web-based nature of this intervention, we were not able to alter the individual’s physical environment and therefore did not target physical opportunity. Capability, opportunity, and motivation were targeted through the intervention functions persuasion, education, modeling, enablement, environmental restructuring (restructuring of intervention platform), and training. Additionally, 13 BCTs from the BCT taxonomy v1 were chosen to deliver the intervention content.

### Utility of a Theoretical Framework

Informing behavioral interventions by theory not only provides a means to increase the efficacy of these interventions, but also allows researchers to standardize reporting of the active ingredients of interventions through BCTs. Current guidelines for reporting behavioral interventions are largely focused on reporting intervention delivery rather than intervention content [29,30]. Consequently, few reports detail active components of existing behavioral interventions and often use different language to describe active components. This presents a barrier to evaluating and replicating aspects of interventions that effectively bring about behavioral change. Experts in behavioral medicine have reported a low level of confidence in their ability to replicate effective behavioral interventions, which is likely linked to poor reporting of these interventions [19]. The current intervention is among a small number of multicomponent behavioral interventions to report on theoretically informed intervention development in a standardized manner [30]. In addition, this is the first known mind-body intervention tailored to PBC. This report provides a basis for (1) better consensus to be reached around a standardized approach to employing behavior change theory to inform an intervention and (2) evidence to be synthesized around which BCTs are effective in the context of an intervention. Both of these factors will allow for replication of successful aspects of implementation and successful active components. Importantly, after study rollout is complete, subsequent qualitative and quantitative assessment of behavior change will be necessary to determine successful components of the intervention.

### Limitations

This project is not without limitations that should be acknowledged. The qualitative feedback used to inform barriers and facilitators to participating in the intervention was provided by participants with IBD, with no large-scale data collection occurring from individuals with PBC. Given the similarity of the symptom burden experienced with IBD and PBC (eg, fatigue, depression, anxiety, stress) the barriers and facilitators provided in the interviews were deemed to be applicable to PBC. To further mitigate this limitation, we worked with an advisory team of patients with PBC to better understand how intervention design needed to be tailored to meet the specific needs of this population (eg, providing a chair stream within the mindful movement to accommodate for potential fatigue and mobility restrictions).

### Conclusions

To our knowledge, the PPP_PBC_ intervention is unique in that it is a mind-body wellness program designed for individuals with PBC, and in that it has taken a structured approach to considering theory in design and evaluation. Development was informed by the BCW [16] and BCTs [19]. Application of these frameworks was guided by feedback from our patient advisory team. Further standardized reporting of complex interventions conducted in different contexts, along with subsequent assessment of behavior change, is necessary to determine how contextual variables influence the effectiveness of different BCTs. 

## Appendix

Multimedia Appendix 1 Description of the web-based intervention.

## Abbreviations

BCT: behavior change technique
BCTv1: behavior change technique taxonomy version 1
BCW: Behaviour Change Wheel
COM-B: Capability, Opportunity, Motivation and Behaviour
HRQOL: health-related quality of life
IBD: inflammatory bowel disease
PBC: primary biliary cholangitis
PPP_PBC_: Peace Power Pack PBC
TDF: theoretical domains framework

## References

[1] (2017). Clinical Review Report: Obeticholic Acid (Ocaliva): (Intercept Pharmaceuticals Canada, Inc.). Ottawa (ON): Canadian Agency for Drugs and Technologies in Health.

[2] Khanna A, Hegade V, Jones D (2019). Management of Fatigue in Primary Biliary Cholangitis. Curr Hepatology Rep.

[3] Reshetnyak VI (2015). Primary biliary cirrhosis: Clinical and laboratory criteria for its diagnosis. World J Gastroenterol.

[4] Witt-Sullivan H, Heathcote J, Cauch K, Blendis L, Ghent C, Katz A, Milner R, Pappas SC, Rankin J, Wanless IR (1990). The demography of primary biliary cirrhosis in Ontario, Canada. Hepatology.

[5] Lee JY, Danford CJ, Trivedi HD, Tapper EB, Patwardhan VR, Bonder A (2019). Treatment of Fatigue in Primary Biliary Cholangitis: A Systematic Review and Meta-Analysis. Dig Dis Sci.

[6] Mells GF, Pells G, Newton JL, Bathgate AJ, Burroughs AK, Heneghan MA, Neuberger JM, Day DB, Ducker SJ, Sandford RN, Alexander GJ, Jones DEJ, UK-PBC Consortium (2013). Impact of primary biliary cirrhosis on perceived quality of life: the UK-PBC national study. Hepatology.

[7] Cauch-Dudek K, Abbey S, Stewart DE, Heathcote EJ (1998). Fatigue in primary biliary cirrhosis. Gut.

[8] Rudic JS, Poropat G, Krstic MN, Bjelakovic G, Gluud C (2012). Ursodeoxycholic acid for primary biliary cirrhosis. Cochrane Database Syst Rev.

[9] Carey EJ, Ali AH, Lindor KD (2015). Primary biliary cirrhosis. Lancet.

[10] Shohani M, Kazemi F, Rahmati S, Azami M (2020). The effect of yoga on the quality of life and fatigue in patients with multiple sclerosis: A systematic review and meta-analysis of randomized clinical trials. Complement Ther Clin Pract.

[11] Araujo RV, Fernandes AFC, Nery IS, Andrade EMLR, Nogueira LT, Azevedo FHC (2019). Meditation effect on psychological stress level in women with breast cancer: a systematic review. Rev Esc Enferm USP.

[12] Izgu N, Gok Metin Z, Karadas C, Ozdemir L, Metinarikan N, Corapcıoglu D (2020). Progressive Muscle Relaxation and Mindfulness Meditation on Neuropathic Pain, Fatigue, and Quality of Life in Patients With Type 2 Diabetes: A Randomized Clinical Trial. J Nurs Scholarsh.

[13] Jennings HM, Morrison J, Akter K, Kuddus A, Ahmed N, Kumer Shaha S, Nahar T, Haghparast-Bidgoli H, Khan AA, Azad K, Fottrell E (2019). Developing a theory-driven contextually relevant mHealth intervention. Glob Health Action.

[14] Cotter AP, Durant N, Agne AA, Cherrington AL (2014). Internet interventions to support lifestyle modification for diabetes management: a systematic review of the evidence. J Diabetes Complications.

[15] Hagger MS, Weed M (2019). DEBATE: Do interventions based on behavioral theory work in the real world?. Int J Behav Nutr Phys Act.

[16] Michie S, van Stralen MM, West R (2011). The behaviour change wheel: a new method for characterising and designing behaviour change interventions. Implement Sci.

[17] Robinson E, Higgs S, Daley AJ, Jolly K, Lycett D, Lewis A, Aveyard P (2013). Development and feasibility testing of a smart phone based attentive eating intervention. BMC Public Health.

[18] Mangurian C, Niu GC, Schillinger D, Newcomer JW, Dilley J, Handley MA (2017). Utilization of the Behavior Change Wheel framework to develop a model to improve cardiometabolic screening for people with severe mental illness. Implement Sci.

[19] Michie S, Richardson M, Johnston M, Abraham C, Francis J, Hardeman W, Eccles MP, Cane J, Wood CE (2013). The behavior change technique taxonomy (v1) of 93 hierarchically clustered techniques: building an international consensus for the reporting of behavior change interventions. Ann Behav Med.

[20] Cane J, O'Connor D, Michie S (2012). Validation of the theoretical domains framework for use in behaviour change and implementation research. Implement Sci.

[21] Watt M, Hyde A, Madsen K, Peerani F, Tandon P (2021). A169 exploring patient perspectives on an online stress reduction based wellness intervention in patients with inflammatory bowel disease (IBD). J Can Assoc Gastroenterol.

[22] Borren NZ, van der Woude CJ, Ananthakrishnan AN (2019). Fatigue in IBD: epidemiology, pathophysiology and management. Nat Rev Gastroenterol Hepatol.

[23] Knowles SR, Graff LA, Wilding H, Hewitt C, Keefer L, Mikocka-Walus A (2018). Quality of Life in Inflammatory Bowel Disease: A Systematic Review and Meta-analyses-Part I. Inflamm Bowel Dis.

[24] Webster R, Michie S, Estcourt C, Gerressu M, Bailey JV, MenSS Trial Group (2016). Increasing condom use in heterosexual men: development of a theory-based interactive digital intervention. Transl Behav Med.

[25] Tombor I, Shahab L, Brown J, Crane D, Michie S, West R (2016). Development of SmokeFree Baby: a smoking cessation smartphone app for pregnant smokers. Transl Behav Med.

[26] The Wellness Toolbox.

[27] Inouye SK, Bogardus ST, Williams CS, Leo-Summers L, Agostini JV (2003). The role of adherence on the effectiveness of nonpharmacologic interventions: evidence from the delirium prevention trial. Arch Intern Med.

[28] Blackburn P, Freeston M, Baker CR, Jones DEJ, Newton JL (2007). The role of psychological factors in the fatigue of primary biliary cirrhosis. Liver Int.

[29] Wood CE, Hardeman W, Johnston M, Francis J, Abraham C, Michie S (2016). Reporting behaviour change interventions: do the behaviour change technique taxonomy v1, and training in its use, improve the quality of intervention descriptions?. Implement Sci.

[30] Bourne JE, Ivanova E, Gainforth HL, Jung ME (2020). Mapping behavior change techniques to characterize a social cognitive theory informed physical activity intervention for adults at risk of type 2 diabetes mellitus. Transl Behav Med.

