# Supplementary material for: Development of a Theoretically Informed Web-Based Mind-Body Wellness Intervention for Patients With Primary Biliary Cholangitis: Formative Study
Source: JMIR Form Res. 2021 Oct 8;5(10):e29064. doi: 10.2196/29064 (PMC8538029; doi:10.2196/29064)
Supplement: Multimedia Appendix 1 [file formative_v5i10e29064_app1.docx]

**Multimedia Appendix 1:** Description of the online intervention

| **Week number** | **Theme of the week** | **Positive psychology activity of the week** | **Breathwork practices introduced** | **Standing mindful movement postures introduced** | **Chair mindful movement postures introduced** |
| --- | --- | --- | --- | --- | --- |
| **Week 1** | Understanding behaviour change and prioritizing self care | Plan program participation. Brainstorm potential barriers and facilitators to program participation | Shining skull breath,  bellows breath,  full breath,  breath awareness, diaphragmatic breathing, alternate nostril breathing | Cat cow, yoga namaskar, tree, thunderbolt | Marching flow,  mountain pose,  seated oblique crunch,  seated chest press with band,  cow face,  lateral line stepovers,  seated boat pose,  seated hammer curls,  staggered sit to stand,  step backs with support |
| **Week 2** | Connecting with your breath | Select 5 core values |  |  | Seated jacks |
| **Week 3** | Connecting with your emotional body | Complete life wheel activity | Sitali | Yogi squat, yoga namaskar in goddess | Seated skiers |
| **Week 4** | End of program goal setting | Set end of program SMART goals |  | Triangle | Seated skaters |
| **Week 5** | Affirmations and personal power | Select and practice affirmations |  | Seated twist | Standing march with support |
| **Week 6** | Managing thoughts | Learn and practice strategies to manage thoughts |  | Happy baby, reclining twist, wind removing pose | Seated reverse fly |
| **Week 7** | Managing feelings | Learn and practice strategies to manage thoughts |  | Beauty pose | Seated triceps kickbacks |
| **Week 8** | Sleep | Select and practice sleep hygiene tips |  |  | Seated good morning |
| **Week 9** | Self compassion | Complete self-compassion journaling, or choose a self compassion pathway to work on |  | Locust pose | 3 way foot reach |
| **Week 10** | Gratitude | Roll a dice to receive ideas for expressing gratitude |  |  | Staggered stance bent over row |
| **Week 11** | Social connectedness | Build a social support village |  |  | Seated wide leg forward bend with twist |
| **Week 12** | End-of-program review and long term goal setting | Set end of program SMART goals |  |  | Seated toe touches |
